# Diagnosis and Monitoring of Hepatitis B Virus Infection Using the Cobas^®^ HBV Test for Use on the Cobas^®^ 4800 System

**DOI:** 10.3390/microorganisms9030573

**Published:** 2021-03-11

**Authors:** Valérie Ortonne, Mélanie Wlassow, Magali Bouvier-Alias, Giovana Melica, Jean-Dominique Poveda, Syria Laperche, Jean-Michel Pawlotsky, Stephane Chevaliez

**Affiliations:** 1 National Reference Center for Viral Hepatitis B, C and delta, Department of Virology, hôpital Henri Mondor, Université Paris-Est, 94000 Créteil, France; valerie.ortonne@aphp.fr (V.O.); melanie.wlassow@aphp.fr (M.W.); magali.bouvier@aphp.fr (M.B.-A.); jean-michel.pawlotsky@aphp.fr (J.-M.P.); 2 Department of Infectious Diseases, hôpital Henri Mondor, 94000 Créteil, France; giovana.melica@aphp.fr; 3 Laboratoire Cerba, Department of Infectious Diseases, 95310 Cergy-Pontoise, France; jdpoveda@lab-cerba.com; 4 National Institute of Blood Transfusion, Department of Blood-Borne Agents, National Reference Center for Infectious Risks in Transfusion, 75015 Paris, France; slaperche@ints.fr

**Keywords:** hepatitis B virus, HBV DNA, HBV monitoring, real-time PCR, hepatitis B infection diagnosis

## Abstract

(1) Background: Sensitive and accurate nucleic acid amplification technologies are now recommended for hepatitis B virus (HBV) DNA detection and quantification in clinical practice to diagnose and monitor hepatitis B infection. The aim of this study was to assess the analytical and clinical performance of the cobas^®^ HBV Test on the cobas^®^ 4800 System. (2) Methods: Standard panel and clinical specimens were tested in parallel with three different real-time commercial PCR assays including the cobas ^®^ HBV Test, the Cobas^®^ AmpliPrep/Cobas^®^ TaqMan HBV Test v2.0 and Alinity™ *m* HBV assay. (3) Results: The specificity of the cobas^®^ HBV Test was 97.9%. The limit of detection was estimated to be 2.1 IU/mL. Intra-assay and interassay coefficients of variation varied from 0.14% to 1.92% and 2.16% to 12.02%, respectively. HBV DNA levels in patients infected with different HBV genotypes strongly correlated with those measured by the two other commercial comparators assays. (4) Conclusions: The cobas^®^ HBV Test can be confidently used to detect and accurately quantify HBV DNA in clinical practice as well as in clinical trials with the new anti-HBV drugs currently in development.

## 1. Introduction

Chronic hepatitis B virus infection is a global public health problem with an estimated 250 million chronically infected people worldwide [1]. Hepatitis B virus-related end-stage liver disease and hepatocellular carcinoma (HCC) are responsible for more than 700,000 deaths per year [2,3]. Current hepatitis B virus (HBV) treatment is based on the lifelong administration of nucleoside or nucleotide analogues that maintain undetectable HBV DNA levels in the long-term and substantially improve the prognosis of HBV-related liver disease, while decreasing the incidence of their complications, including HCC [4,5]. Another option is a treatment over 48 weeks with pegylated interferon alpha-2a. The ideal treatment goal is the loss of hepatitis B surface antigen (HBsAg) and possible HBs antibody seroconversion, the so-called functional cure of HBV infection.

The use of sensitive nucleic acid amplification technologies for HBV DNA detection and quantification is recommended by international Clinical Practice Guidelines in clinical practice [6,7]. Current HBV assays and analyzer platforms have a broad range of linear quantification and a limit of detection on the order of 10 international unit per milliliter (IU/mL) or less. They should be able both to detect and quantify HBV DNA, regardless of the HBV genotype (with at least 10 HBV genotypes identified) or sequence polymorphisms. The cobas^®^ AmpliPrep/cobas^®^ TaqMan HBV assay, version 2.0 (CAP/CTM HBV Test v2.0; Roche Molecular Systems, Pleasanton, CA, USA) and the Alinity™ *m* HBV assay (Abbott Molecular, Des Plaines, IL, USA) are the most widely used assays in clinical practice. They show satisfactory performance for HBV DNA detection and quantification [8,9,10]. Roche Diagnostics recently introduced the cobas^®^ HBV Test, a polymerase chain reaction (PCR)-based assay that is run on the automated cobas^®^ 4800 System, which allows the delivery of the first 24 results in less than 3 h.

Here, we assessed the ability of the cobas^®^ HBV Test to detect and accurately quantify HBV DNA in a large series of patients infected with HBV genotypes frequently encountered in clinical practice.

## 2. Materials and Methods

### 2.1. Standards

A standard panel containing different concentrations of HBV DNA obtained by dilution of an HBV-positive human in normal human plasma (AcroMetrix™ HBV Panel; Thermo Scientific, Fremont, CA, USA) was used. Each panel member contains a predetermined amount of HBV DNA calibrated against the WHO international standard. The seven panel members, HBV 5E1, HBV 5E2, HBV 5E3, HBV 5E4, HBV 5E5, HBV 5E6, and HBV 5E7 contain 5 × 10^1^ IU/mL (1.70 Log IU/mL), 5 × 10^2^ IU/mL (2.70 Log IU/mL), 5 × 10^3^ IU/mL (3.70 Log IU/mL), 5 × 10^4^ IU/mL (4.70 Log IU/mL), 5 × 10^5^ IU/mL (5.70 Log IU/mL), 5 × 10^6^ IU/mL (6.70 Log IU/mL), and 5 × 10^7^ IU/mL (7.70 Log IU/mL), respectively.

### 2.2. Clinical Specimens

Plasma samples (*n* = 365) were collected from HBsAg-positive and -negative patients in the Department of Hepatology or in the Department Infectious Diseases of the Henri Mondor hospital or in the Cerba laboratory and from HBsAg-positive blood donors at the Institut National de la Transfusion Sanguine. Genotyping based on phylogenetic analysis of a portion of the overlapping S gene was available for 119 clinical specimens including 35 genotype A, 6 genotype B, 10 genotype C, 44 genotype D, 23 genotype E, and 1 genotype G.

The present study was performed in accordance with the principles of Good Clinical Practice and conducted in adherence with the Declaration of Helsinki. Only leftover samples from samples originally sent to our laboratory for routine HBV testing were used. All samples were anonymized prior to the study, with an identification number containing no patient identifiers assigned to each sample.

### 2.3. HBV DNA Quantification

Frozen samples were tested with three different assays, including the real-time PCR-based cobas^®^ HBV Test and two commercially available assays with the CAP/CTM HBV Test v2.0 test and the Alinity™ *m* HBV assay, a PCR-based dual probe assay that is run on a fully automated Alinity *m* analyzer [11].

*cobas^®^ HBV test*: HBV DNA was isolated from 400 µL of plasma using the cobas^®^ 4800 System, which consists of separate devices for sample preparation (cobas × 480) and amplification/detection (cobas z480 analyser). The dynamic range of quantification is 10 to 10^9^ IU/mL (1.0–9.0 Log IU/mL). The limit of detection (LoD) is 4.4 IU/mL in plasma and the lower limit of quantification (LLOQ) is 10 IU/mL. The viral region targeted by the cobas^®^ HBV Test primers and probes is the *preC-C* gene.

*CAP/CTM HBV Test v2.0*: HBV DNA was isolated from 650 µL of plasma using the cobas AmpliPrep and cobas TaqMan 96 analyzer for automated sample extraction and real-time PCR amplification and detection. The dynamic range of quantification is 20 to 1.7 × 10^8^ IU/mL (1.3–8.2 Log IU/mL), with an LoD of 9.0 IU/mL in plasma and an LLOQ of 20 IU/mL. The CAP/CTM HBV Test v2.0 assay primers and probes target the *preC-C* gene.

*Alinity*™ *m HBV assay*: HBV DNA was isolated from 300 µL of plasma on the Alinity *m* System. The dynamic range of quantification is 10 to 10^9^ UI/mL (1.0–9.0 Log IU/mL), with an LoD of 6.4 IU/mL in plasma and an LLOQ of 10 IU/mL. The viral region targeted by Alinity™ *m* HBV assay primers and probes is the *S* gene.

### 2.4. Statistical Analysis

Descriptive statistics are shown as means ± standard deviations (SD) or as medians and interquartile ranges, as appropriate. The LoD of the cobas^®^ HBV Test was determined by means of Logit analysis as the 95%-point estimate with a surrounding 95% confidence interval (95%CI), using the IBM^®^ SPSS^®^ Statistics version 1.0 (Statistical Package for Social Sciences, IBM Corp., Chicago, IL, USA). The relationship between quantitative variables was studied by means of Deming regression analysis. Bland–Altmann plot method was used to evaluate the differences in quantification between the quantitative assays. Comparison between groups was made using the Kruskal–Wallis test. *p* values of <0.05 were considered significant.

## 3. Results

### 3.1. Specificity

To assess the specificity of the cobas^®^ HBV Test, 190 plasma specimens collected from HBsAg-negative subjects were tested. Of the 190 clinical specimens, all tested HBV DNA undetectable except four, which tested HBV DNA detectable but nonquantifiable, resulting in a specificity of 97.9% (95%CI: 94.7−99.2%). The four samples were all except one found HBV DNA negative on retesting with the cobas^®^ HBV Test.

### 3.2. Analytical Sensitivity

To evaluate the analytical sensitivity of the cobas^®^ HBV Test, the AcroMetrix™ HBV Panel was serially diluted from 50 to 1.5625 IU/mL. Twenty replicates of each dilution were tested in the same run, as recommended by the Clinical and Laboratory Standard Institute [12]. The detection rate was 100% for replicates at 50, 25, 12.5, 6.25, and 3.125 IU/mL, and 90% for replicates at 1.5625 IU/mL. The limit of detection, calculated using Logit analysis, was estimated to be 2.10 IU/mL (95%CI was calculable as fiducial limits of the probit curve could not be calculated at the 95% confidence level).

### 3.3. Precision

To assess precision (intra-assay variability), each member of the AcroMetrix™ HBV Panel was tested three times in the same run. The coefficients of variation (CV) varied from 0.14% to 1.92%. To assess the interassay variability, low and high positive controls were tested in 28 different runs. The CVs were 2.16% and 12.02% for the low and high positive controls, respectively.

### 3.4. Linearity of a Standard Panel

**Figure 1:**
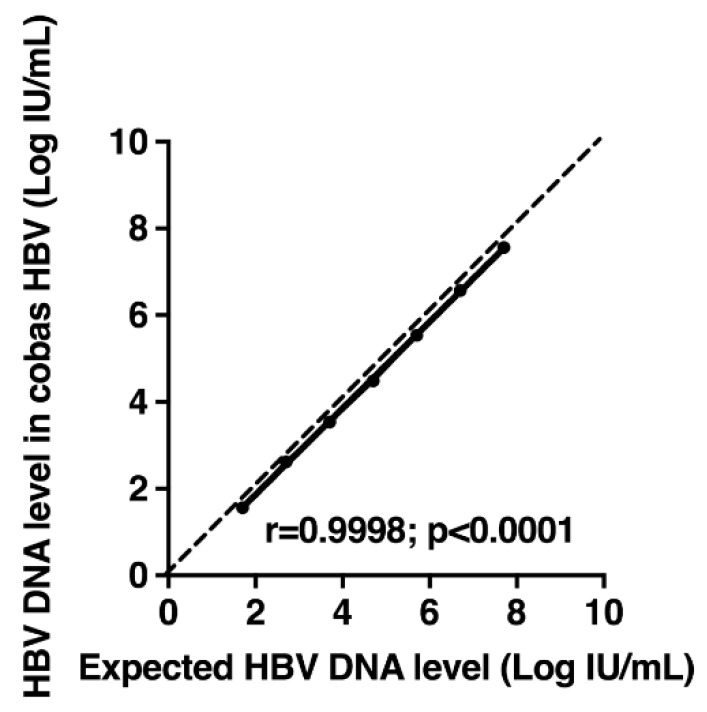
Measurements of hepatitis B virus (HBV) DNA levels in standard panel (AcroMetrix™ HBV Panel; Thermo Scientific, Fremont, CA, USA) containing different concentrations of HBV DNA with the cobas^®^ HBV Test. The average measured values are shown as a function of the expected values. The dashed line represents the equality line.

To evaluate the linearity of HBV DNA quantification, each member of the AcroMetrix™ HBV Panel was tested three times with the cobas^®^ HBV Test, and the measured values were compared to the expected ones. A significant correlation was observed between the average measured and expected HBV DNA levels (r = 0.9998; *p* < 0.0001) (Figure 1). Differences between the average measured values and the expected values varied from −0.21 to −0.08 Log IU/mL.

### 3.5. Influence of Genotype on HBV DNA Quantification

To study the influence of the genotype on HBV DNA quantification, samples from HBsAg-positive patients infected with different HBV genotypes were tested in parallel with cobas^®^ HBV Test and one of the two comparator assays: CAP/CTM HBV Test v2.0 (*n* = 121) and Alinity™ *m* HBV assay (*n* = 83).

**Figure 2:**
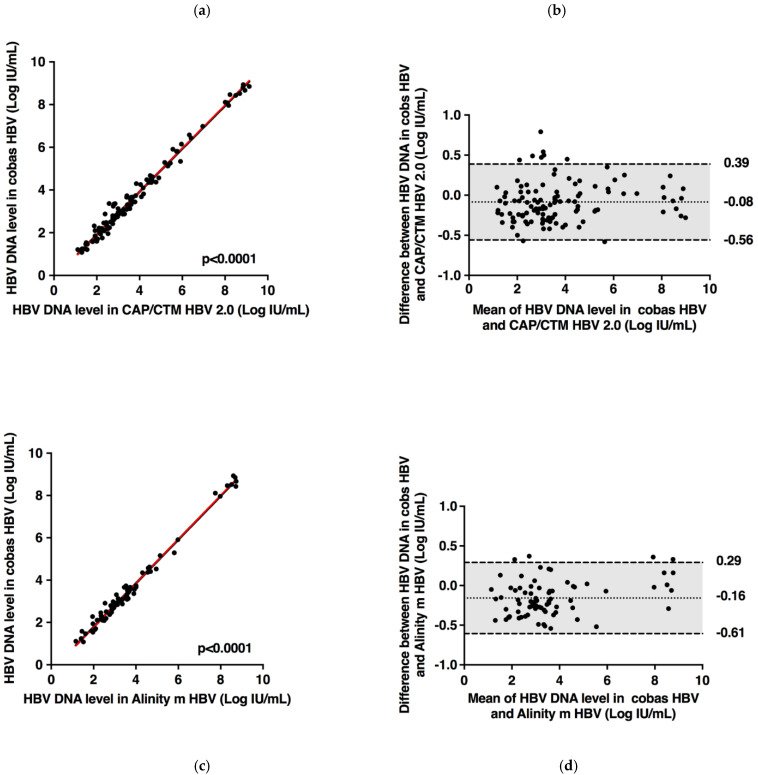
Deming linear regression (**a**) and Bland–Altman plot analysis (**b**) of HBV DNA measured by cobas^®^ HBV Test and CAP/CTM HBV Test v2.0 in 121 plasma specimens containing different HBV genotypes. Deming linear regression (**c**) and Bland–Altman plot analysis (**d**) of HBV DNA measured by cobas^®^ HBV Test and Alinity™ *m* HBV assay in 83 plasma specimens containing different HBV genotypes. In the Bland-Altman graphs, the dotted and dashed lines represent the mean difference and the ±1.96 SD, respectively.

Of the 121 samples tested by the cobas^®^ HBV Test and CAP/CTM HBV Test v2.0, all fell within the dynamic range of both assays. A strong correlation between the two assays was found (r = 0.992, Deming regression equation, y = 1.016 × −0.145) (Figure 2). A weak bias across the quantitative range of the two assays was observed using the Bland–Altman analysis (0.08 ± 0.24 Log IU/mL), indicating no major difference in quantification according to both HBV DNA level and genotype. In 92.6% (112/121) of the plasma specimens, the difference between the assays was within 1.96 times the SD of bias. In 7.4% (9/121) of specimens where the difference exceeded 1.96 times the SD of bias, 5.8% (7/121) of results were higher and 1.6% (2/121) were lower than 1.96 times the SD of bias with the cobas^®^ HBV Test compared to the CAP/CTM HBV Test v2.0.

Of the 83 samples tested by the cobas^®^ HBV Test and Alinity™ *m* HBV assay, all fell within the dynamic range of both assays. A strong correlation between the two assays was found (r = 0.994, Deming regression equation, y = 1.039 × −0.3074) (Figure 2). Bland–Altman analysis demonstrated a weak bias across the dynamic range of the two assays (−0.16 ± 0.23 Log IU/mL). No major difference in quantification was observed according to both HBV DNA level and genotype. In 95.2% (79/83) of the plasma specimens, the difference between the assays was within 1.96 times the SD of bias. In 4.8% (4/83) of specimens, the difference was systematically higher than 1.96 times the SD of bias with the cobas^®^ HBV Test compared to the Alinity™ *m* HBV assay.

**Figure 3:**
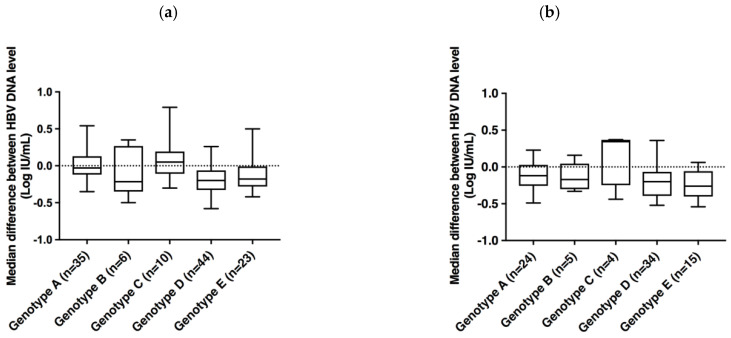
Box plot representation of the distribution of the median differences between HBV DNA levels measured by the cobas^®^ HBV Test and CAP/CTM HBV Test v2.0 (**a**) or Alinity™ *m* HBV assay (**b**).

Figure 3 shows individual differences between the cobas^®^ HBV Test and the two comparators for each genotype. That confirms an overall moderate underestimation of HBV DNA levels in cobas^®^ HBV Test as compared to both comparators, regardless of the HBV genotype. The median difference between the cobas^®^ HBV Test and CAP/CTM HBV Test v2.0 was −0.03 Log IU/mL for genotype A, −0.21 Log UI/mL for genotype B, 0.05 Log IU/mL for genotype C, −0.2 Log IU/mL for genotype D, and −0.18 Log IU/mL for genotype E. The median difference between the cobas^®^ HBV Test and Alinity™ *m* HBV assay was −0.12 Log IU/mL for genotype A, −0.17 Log UI/mL for genotype B, 0.34 Log IU/mL for genotype C, −0.2 Log IU/mL for genotype D, and −0.26 Log IU/mL for genotype E.

### 3.6. Overall Percent Agreement for the Clinical Cutoffs of 2000 and 20,000 UI/mL

Table 1 and Table 2 show the overall percent agreement between the cobas^®^ HBV Test and the two comparator assays (CAP/CTM HBV Test v2.0 and Alinity™ *m* HBV assay) for the two clinically relevant cutoffs of 2000 and 20,000 UI/mL, respectively.

At the 2000 UI/mL cutoff, the overall percent agreements between the cobas^®^ HBV Test and the CAP/CTM HBV Test v2.0 or Alinity™ *m* HBV assay were 92.6% and 92.8%, respectively. At the 20,000 UI/mL cutoff, the overall percent agreements between the cobas^®^ HBV Test and the CAP/CTM HBV Test v2.0 or Alinity™ *m* HBV assay were 99.2% and 100%, respectively.

### 3.7. HBV DNA Monitoring in Patients Treated with Tenofovir-Containing Regimen

**Figure 4:**
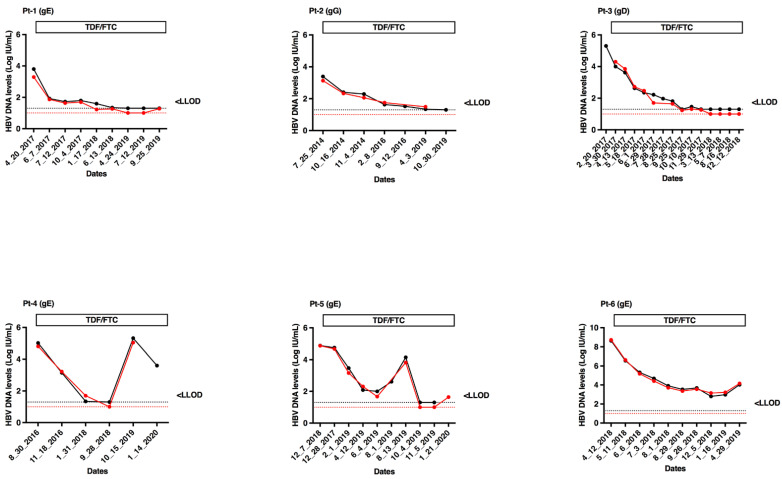
Kinetics of HBV DNA levels measured with cobas^®^ HBV Test (red lines and filled circles) and CAP/CTM HBV Test v2.0 (black lines and filled circles) in treated patients with tenofovir-containing regimens. The red and black dashed lines correspond to the LLOQ of cobas^®^ HBV Test (1.0 Log IU/mL) and CAP/CTM HBV Test v2.0 (1.3 Log IU/mL), respectively. Pt, patient; TDF, tenofovir; FTC, emtricitabine; gE, genotype E; gG, genotype G; gD, genotype D.

Six patients chronically coinfected with HBV (one with genotype D, four with genotype E and one with genotype G) and HIV were serially sampled during treatment with tenofovir and emtricitabine. Fifty-four specimens were tested in parallel with the cobas^®^ HBV Test and CAP/CTM HBV Test v2.0. In all cases, HBV DNA dynamics were strictly parallel (Figure 4), demonstrating a satisfactory concordance between the two assays.

## 4. Discussion

Sensitive and accurate nucleic acid amplification technologies are recommended by international Clinical Practice Guidelines for the quantification and detection of HBV DNA in clinical practice [6,7]. Measurements of HBV DNA level are essential to diagnose HBV infection, establish the prognosis of HBV related liver disease, and guide the decision to treat and monitor the virological response to antiviral treatment and the emergence of resistance.

The present study, based on a large series of clinical specimens including samples with different HBV genotypes frequently encountered in clinical practice, showed that the cobas^®^ HBV test accurately quantified HBV DNA in plasma. Its performance was in keeping with that of other real-time PCR or TMA-based platforms and assays frequently used in clinical practice [11,13,14,15]. The cobas^®^ HBV Test was very sensitive with an estimated LoD on the same order as that claimed by the manufacturer. Interestingly, the cobas^®^ HBV test showed a slightly improved sensitivity compared with other HBV assays, as previously demonstrated [16]. This assay was precise and results were reproducible, as previously reported [17,18,19]. We observed only a modest difference in HBV DNA levels when compared with the CAP/CTM HBV Test v2.0 (0.08 ± 0.24 Log IU/mL) and Alinity™ *m* HBV assay (−0.16 ± 0.23 Log IU/mL), respectively. This finding was independent of the HBV genotype and HBV DNA level and has most likely no clinical implications, as shown when serial samples from patients receiving nucleoside or nucleotide analogues were tested. Indeed, HBV DNA kinetics in patients receiving antiviral treatment showed a perfect concordance of the results between assays. In addition, concordance around important clinical decision points, including the 2000 and 20,000 IU/mL thresholds, was generally high between all three HBV assays. Not surprisingly, the risk for discordant results increased if the HBV DNA level was closed to the 2000 IU/mL or the 20,000 IU/mL cutoff, as also documented previously [16].

Our study has several limitations. The number of clinical specimens collected in HBsAg-positive patients is relatively small. The proportion of samples containing genotypes B and C is not as important as other genotypes. This reflects the HBV genotype distribution in our country, where genotypes A, D and E are the most prevalent. Few specimens containing rare genotypes (F, G, H, and I) were included. Finally, not all included plasma specimens could be tested by three molecular assays, due to an insufficient volume in some of them.

## 5. Conclusions

The present study showed that the cobas^®^ HBV Test is sensitive, specific, reproducible, and accurately quantifies HBV DNA in plasma specimens from patients with chronic HBV infection, including patients receiving nucleotide or nucleoside analogues. The cobas^®^ HBV Test can be confidently used to detect and quantify HBV DNA in clinical practice as well as in clinical trials with the new anti-HBV drugs currently in development.

## Figures and Tables

**Table 1:** Concordance at the 2000 and 20,000 UI/mL clinically relevant cutoffs between the cobas^®^ HBV test and CAP/CTM HBV Test v2.0.

		CAP/CTM HBV Test v2.0		
**cobas^®^ HBV Test**		<2000 IU/mL	≥2000 IU/mL	Total
	<2000 IU/mL	55	6	61
	≥2000 IU/mL	3	57	60
	Total	58	63	121
		<20,000 IU/mL	≥20,000 IU/mL	Total
	<20,000 IU/mL	84	0	84
	≥20,000 IU/mL	1	36	37
	Total	85	36	121

**Table 2:** Concordance at the 2000 and 20,000 UI/mL clinically relevant cutoffs between the cobas^®^ HBV test and Alinity™ *m* HBV assay.

		Alinity™ *m* HBV		
**cobas^®^ HBV Test**		<2000 IU/mL	≥2000 IU/mL	Total
	<2000 IU/mL	39	5	44
	≥2000 IU/mL	1	38	39
	Total	40	43	83
		<20,000 IU/mL	≥20,000 IU/mL	Total
	<20,000 IU/mL	62	0	62
	≥20,000 IU/mL	0	21	21
	Total	62	21	83

## Data Availability

The data presented in this study are available from the authors on request.
